# Seaweed Proteins: Properties, Extraction, Challenges, and Prospects

**DOI:** 10.1111/1750-3841.70418

**Published:** 2025-07-21

**Authors:** Sakhi Ghelichi, Charlotte Jacobsen

**Affiliations:** ^1^ Research Group for Bioactives – Analysis and Application, National Food Institute Technical University of Denmark Lyngby Denmark

**Keywords:** bioactive properties, cell wall, extraction methods, nutritional aspects, protein purification, protein quantification, seaweed protein

## Abstract

The growing global population and increasing demand for sustainable protein sources have intensified interest in seaweed as a viable plant‐based alternative. Seaweed‐derived proteins and peptides are rich in essential amino acids and exhibit promising functional and bioactive properties, making them attractive for food applications. However, several challenges hinder their widespread use, including difficulties in protein extraction and purification, the presence of potentially harmful compounds, and limited digestibility of the extracted proteins. This review critically examines the nutritional, functional, and bioactive properties of proteins and peptides derived from seaweed, while also addressing key obstacles related to extraction and application. Specific focus is placed on issues such as the rigidity of the seaweed cell wall, low protein solubility, digestibility, and the presence of antinutritional factors, toxic compounds, and allergens. Given that protein quantification remains a significant challenge in this field, current quantification methods and their limitations are also reviewed. Furthermore, this study provides a critical evaluation of the extraction methods employed for isolating proteins and peptides from seaweed. This review also emphasizes the importance of adopting a biorefinery perspective for seaweed protein extraction to ensure the efficient and sustainable utilization of the entire seaweed biomass. Moreover, this review examines the ongoing commercial development of seaweed protein and its potential for market integration. In conclusion, future perspectives are discussed, including the potential of bottom‐up approaches, the integration of seaweed‐derived proteins and peptides into food systems, and the importance of understanding their interactions with other biomolecules, which may influence their functional and nutritional properties.

## 1 Introduction

The growing global concern over the environmental consequences of livestock farming, particularly its contribution to greenhouse gas emissions, deforestation, and excessive water consumption, has intensified the search for more sustainable protein sources (Gil et al. 2024). Algae‐based proteins, particularly those derived from seaweed, present a highly sustainable and nutritionally rich alternative to animal‐derived proteins. Algae cultivation is characterized by rapid growth, minimal water usage, and no competition for arable land, making it an environmentally efficient protein source (Karabulut et al. 2024). In this regard, seaweed proteins and peptides, with their functional properties like thickening and stabilizing, and bioactive properties such as antioxidant, antihypertensive, and antimicrobial effects, are promising candidates for both nutritional and therapeutic applications, particularly in plant‐based diets (Pereira et al. 2024). Seaweeds, classified into red (Rhodophyta), brown (Phaeophyceae), and green (Chlorophyta) based on their pigments, vary in protein content depending on species, seasonality, and environment. Red seaweeds generally have the highest levels, and several protein‐rich varieties, such as *Ulva lactuca* (Chlorophyta), *Undaria pinnatifida* (Phaeophyceae), and *Palmaria palmata* (Rhodophyta), are approved for human consumption by the European Food Safety Authority (de Souza Celente et al. 2023).

Despite its advantages, extracting protein from seaweed presents several technical and scientific challenges that must be addressed to realize its full potential. The rigid and complex structure of seaweed cell walls, composed of cross‐linked proteins and other biopolymers like polysaccharides, creates a significant barrier to the efficient release of intracellular proteins (Naz and Mukherjee 2025). Additionally, seaweed proteins often interact with other biomolecules, such as lipids, polysaccharides, and phenolic compounds, which can complicate the extraction process and affect the purity of the final product (Juel et al. 2024). Furthermore, the quantification of protein content in seaweed can be problematic due to the presence of nonprotein nitrogen compounds, leading to discrepancies in the estimation of protein yields (Samarathunga et al. 2023).

Various techniques are employed to extract protein from seaweed, including conventional methods like alkaline (Juul et al. 2023), acid (Ghelichi, Hajfathalian, et al. 2025), enzymatic (Teixeira‐Guedes et al. 2023), and mechanical and physical extraction (Naseem et al. 2024b), as well as green technologies such as microwave digestion (Wijethunga et al. 2025), pulsed electric field (Maribu et al. 2024), ultrasound‐assisted extraction (Yücetepe et al. 2024), and subcritical water treatment (Trigueros et al. 2021). Each of these methods has distinct strengths and limitations regarding yield, protein quality, cost, and sustainability. This variation highlights the need to evaluate their efficiency to identify the efficient methods for large‐scale production and applications.

In addition to extraction challenges, the nutritional quality of seaweed proteins must also be considered. Although seaweed proteins are generally rich in essential amino acids (EAAs), they may lack certain key nutrients such as histidine and methionine found in other protein sources, necessitating the mixing of proteins from different seaweed species (Reynolds et al. 2022). Moreover, factors such as protein digestibility and the presence of antinutritional factors might affect the bioavailability of seaweed protein in human diets (Landeta‐Salgado et al. 2025). These issues necessitate further research to optimize protein extraction methods and enhance the nutritional profile of seaweed‐based protein products.

This review focuses on the nutritional and bioactive properties of seaweed proteins while exploring the challenges and potential of their extraction. It addresses key factors such as rigid cell walls, protein interactions with biomolecules, and inaccuracies in protein quantification, including the nitrogen‐to‐protein conversion factor. Various extraction methods, including chemical, enzymatic, mechanical, and green technologies, are assessed for their effectiveness across different seaweed species. Additionally, the review highlights the significance of integrating seaweed protein extraction into biorefinery processes to enhance sustainability and resource efficiency and explains the current status and prospect of seaweed protein in terms of its marketability and commercial development. The review concludes by summarizing key insights and outlining prospects for seaweed protein extraction.

## 2 Nutritional, Functional, and Bioactive Properties of Seaweed Protein

### 2.1 Nutritional Properties

The nutritional quality of seaweed protein is primarily determined by its amino acid composition, with a complete profile of EAAs playing a crucial role in its value as a dietary protein source (Reynolds et al. 2022). The protein content across different seaweed species varies, but many offers significant amounts of EAAs, contributing to their high nutritional potential (Premarathna et al. 2022). While many plant‐based proteins, such as those from soy and pea, have incomplete amino acid profiles, seaweed protein stands out as a promising alternative for developing high‐quality proteins (Rawiwan et al. 2022). Table 1 summarizes the total content and amino acid composition of proteins extracted from some seaweed species, as well as the limiting EAA(s) in these proteins. As illustrated in the table, the nutritional value of protein extracted from seaweed is significantly influenced by both the species of seaweed and the extraction method employed. Various species contain different protein profiles, which can impact the overall quality and composition of the extracted protein. Additionally, seasonal variations play a crucial role in altering the nutritional properties of seaweed‐derived proteins. Studies have shown that the timing of harvest can lead to notable changes in the protein content and quality, as environmental factors, such as temperature and nutrient availability, fluctuate throughout the year (Aamiri et al. 2024; Arlov et al. 2024). This emphasizes the importance of considering both species‐specific and temporal factors when assessing the nutritional value of seaweed protein. Moreover, it is important to exercise caution when evaluating the protein content of seaweed extracts in relation to the nitrogen‐to‐protein conversion factor. Some studies may have overestimated protein content by applying a conversion factor of 6.25, which has been shown to significantly overestimate protein levels in seaweed (Bjarnadóttir et al. 2018). A more detailed discussion of this topic is provided later in this review.

**TABLE 1:** Total protein content, essential amino acids, and limiting essential amino acids in extracts from some seaweed species.

Seaweed species	Type of seaweed	Extraction method	Total protein content (%)	Nitrogen‐to‐protein conversion factor	Essential amino acids (%)	Limiting essential amino acids	Reference
*Chondrus crispus*	Red	Mechanical	19.5 ± 0.16	6.25	46.7	Met	Vieira et al. 2018
*Alaria esculenta*	Brown	Sonication/salting out	18.2 ± 5.16	4.45	41.99	His	O'Connor et al. 2020
*Himanthalia elongata*	Brown	Sonication/chemical	6.5 ± 0.7	6.25	∼40	Met	Garcia‐Vaquero et al. 2017
*Saccharina latissima*	Brown	Chemical	∼25	L^a^	42.6	His–Met	Harrysson et al. 2018
*Eucheuma denticulatum*	Red	Enzymatic/alkaline	28.6 ± 0.44	5	∼38.5	His–Met	Gregersen et al. 2021
*Ulva* sp.	Green	Enzymatic/pulsed electric field	13.4	L	40.7	His–Met	Steinbruch et al. 2024
*Palmaria palmata*	Red	Alkaline	6.96 ± 0.20	5	39.75	His	Ghelichi, Hajfathalian, et al. 2025
*Palmaria palmata*	Red	Enzymatic/alkaline	11.20 ± 0.16	5	44.03	His	Ghelichi, Sørensen, Hajfathalian, et al. 2024
*Ascophyllum nodosum*	Brown	Microwave/enzymatic/supercritical CO_2_	3.39 ± 0.06	6.25	4.65	N/A^b^	Periaswamy Sivagnanam et al. 2024
*Ulva compressa*	Green	Mechanical/chemical	29.5	5.4	40.1	His	Nissen et al. 2024

^a^ Lowry method was employed for protein analysis, and as such, a nitrogen‐to‐protein conversion factor was not applied.

^b^ Not applicable, as the content of essential amino acids is negligible, with most of them being absent.

In terms of amino acid composition, seaweed‐derived protein generally contains approximately 40% EAAs relative to total amino acids, except in low‐protein species. This composition makes seaweed a valuable protein source, particularly as a plant‐based alternative. However, as shown in Table 1, methionine and histidine are the limiting EAAs in seaweed protein, a factor that must be considered for nutritional applications. Recent studies have also highlighted variations in EAA content among different seaweed species, further emphasizing the need for species‐specific analysis (Landeta‐Salgado et al. 2025). Compared to animal‐based proteins, which are typically complete proteins containing all EAAs in sufficient amounts (Azizi et al. 2024), seaweed protein may require supplementation with other protein sources to achieve optimal nutritional value. While seaweed protein offers a promising EAA profile as an alternative protein source, its methionine and histidine limitations suggest that it is best utilized in combination with complementary protein sources to ensure a balanced amino acid intake for human nutrition.

Beyond protein content and amino acid composition, digestibility is crucial for evaluating seaweed protein quality. The Protein Digestibility‐Corrected Amino Acid Score (PDCAAS) was introduced to assess protein quality by multiplying the limiting amino acid score by protein digestibility, aiming to determine how well dietary protein meets amino acid requirements. However, given the limitations of PDCAAS, the Digestible Indispensable Amino Acid Score (DIAAS) has been recommended as a more accurate method. This measure calculates protein quality based on the true ileal digestibility of each indispensable amino acid, providing a better assessment of bioavailable protein (FAO 2013). The method applied to assess protein quality may also differ by country, especially in relation to regulatory frameworks and labeling standards. For example, PDCAAS remains the accepted standard in countries like the United States for determining protein quality in nutrition labeling, while DIAAS has been recommended by the Food and Agriculture Organization (FAO) for more accurate assessment, although it is not yet widely adopted in regulatory contexts (Mansilla et al. 2020). A recent study reported PDCAAS values for some brown (*Durvillaea* spp. and *Macrocystis pyrifera*) and green (*Ulva* spp.) seaweed species, as well as for a mycoprotein extracted from *Durvillaea* spp. The seaweed showed relatively low PDCAAS scores of 0.53, 0.41, and 0.37, respectively, with correspondingly low DIAAS values, indicating limited protein quality without processing. In contrast, the extracted mycoprotein demonstrated a high PDCAAS score of 0.89 and superior nutritional quality, particularly for lysine and histidine as highlighted by DIAAS values (Landeta‐Salgado et al. 2025). Similarly, red and brown seaweeds such as *P. palmata*, *Fucus serratus*, and *Alaria esculenta* exhibited moderate k‐PDCAAS values of 0.69, 0.63, and 0.59, respectively (Moreno et al. 2022). The application of the in vivo methods to assess seaweed protein digestibility is relatively recent, and current knowledge is largely limited to in vitro studies. Despite their limitations, these in vitro data provide a useful baseline for comparing the digestibility of seaweed proteins with that of more conventional protein sources (Reynolds et al. 2022).

### 2.2 Functional Properties

Seaweed can be considered a valuable source of protein with various functional properties, including solubility, emulsification, foaming, gelling, and the ability to bind water and oil. The solubility of seaweed proteins varies depending on the species and extraction method. Generally, seaweed proteins exhibit favorable solubility in alkaline conditions, as demonstrated for brown seaweed *Saccharina latissima* (Abdollahi et al. 2019) and *Himanthalia elongata* (Garcia‐Vaquero et al. 2017), red seaweed *P. palmata* (Ghelichi, Sørensen, Hajfathalian et al. 2024) and *Kappaphycus alvarezii* (Suresh Kumar et al. 2014), and green seaweed species of *Ulva* (formerly *Enteromorpha*) (Kandasamy et al. 2012), which is indicative of their potential use in liquid‐based food products such as beverages, soups, and sauces. However, high pH values can adversely affect the sensory properties of the proteins, resulting in darker colors and unpleasant flavors, and may also disrupt EAAs (Yang et al. 2023). These changes may reduce consumer acceptance and limit the application of seaweed proteins in food products. Therefore, a balance between protein solubility and alkalinity must be maintained for optimal functionality.

Seaweed proteins have also demonstrated emulsifying activity, which makes them potential candidates for use in emulsion‐based food products like dressings, sauces, and spreads. For instance, Abdollahi et al. (2019) reported that proteins obtained from *S. latissima* exhibited favorable emulsifying properties at both neutral and alkaline pH of the emulsion, as indicated by an emulsion activity index (EAI) of >40 m^2^/g. However, the ability of these proteins to stabilize emulsions was significantly higher under alkaline conditions of the emulsion, as demonstrated by increased values of the emulsion stability index (ESI) when using both oven‐dried and freeze‐dried biomass. Yesiltas et al. (2023) showed that three peptides synthesized based on the amino acid sequences of peptides derived from seaweed protein can physically stabilize fish oil‐in‐water emulsions, as evidenced by metrics such as zeta potential, mean droplet diameter (volume and surface moment), and observations of creaming stability. Despite the potential of seaweed‐derived protein‐rich fractions as emulsifying agents, studies on their effectiveness in food applications remain limited in the literature. Therefore, future research should focus on exploring the emulsifying properties of these fractions in both model and real food systems. Additionally, comparative studies evaluating the emulsifying performance of seaweed proteins against commercial emulsifiers, such as sodium caseinate, lecithin, and Tween, are essential to fully understand their potential for food applications.

In addition to solubility and emulsifying properties, seaweed proteins exhibited foaming as well as water‐ and oil‐holding properties. For example, Felix et al. (2021) reported that protein concentrate from *Porphyra dioica* (Rhodophyta) exhibited foaming properties comparable to commercial proteins, as indicated by its overall foam capacity of >0.7 mL/s. However, it was less effective than commercial stabilizers in enhancing foam stability. In addition, Garcia‐Vaquero et al. (2017) reported that protein concentrate from *H. elongata* exhibited favorable foaming capacity at neutral and alkaline pH of the protein suspensions, but not in acidic conditions, likely due to charge‐induced changes that reduced hydrophobic interactions, increased protein flexibility, and improved migration to the air–water interface, facilitating foam formation. They also observed greater foam stability under alkaline conditions. Therefore, seaweed‐derived protein shows promise as a food‐grade, nonanimal protein for use in dispersed systems. Furthermore, proteins from various seaweed species, such as *K. alvarezii* (Suresh Kumar et al. 2014) and *H. elongata* (Garcia‐Vaquero et al. 2017), have demonstrated water‐ and oil‐holding capacity, highlighting their potential to enhance the textural and flavor properties of foods. Overall, the functional properties of seaweed proteins, including solubility, emulsification, foaming, and water‐ and oil‐holding capacity, emphasize their potential as versatile ingredients for improving the texture, stability, and sensory characteristics of food products.

### 2.3 Bioactive Properties

Several studies have explored the bioactive properties of seaweed‐derived proteins and peptides. Given the scope of this review and space limitations, an in‐depth discussion of these studies is not provided here. However, a brief overview of these effects is presented in this section to emphasize the potential of seaweed proteins and the importance of efforts to extract protein from seaweed. Table 2 provides examples of the bioactive properties of seaweed‐derived proteins. As summarized in the table, proteins and peptides derived from various seaweed species have demonstrated a wide range of bioactive properties, including antioxidant (Windarto et al. 2024), antihypertensive (Purcell et al. 2022), antimicrobial (Hardouin et al. 2016), antiobesity and antidiabetic (Ghelichi, Hajfathalian, et al. 2025), anti‐inflammatory and immunomodulatory (Sanniyasi et al. 2023), and antiproliferative, antitumor, and anticancer (Du et al. 2022) effects. These findings highlight the promising potential of seaweed as a nonanimal source of bioactive proteins and peptides with applications in functional foods, nutraceuticals, and pharmaceuticals (Matos et al. 2024). However, despite this promising potential, the current body of research on the bioactive properties of seaweed‐derived proteins and peptides remains relatively limited. While several studies have explored these effects, further investigations are needed to fully understand their mechanisms of action, optimize extraction and purification techniques, and assess their bioavailability and efficacy in human models. Expanding research in this area could unlock new possibilities for utilizing seaweed‐derived proteins and peptides as sustainable alternatives to conventional bioactive compounds.

**TABLE 2:** Bioactive properties of proteins and peptides derived from various seaweed species.

Bioactivity	Seaweed species	Seaweed type	Extraction method	Evaluation method	Reference
Antioxidant	*Kappaphycopsis cottonii* (formerly *Eucheuma cottonii*)	Red	Enzymatic hydrolysis	In vitro radical scavenging	Sun et al. 2022
	*Gracilariopsis lemaneiformis*	Red	Enzymatic hydrolysis	In vitro radical scavenging	Zhang et al. 2019
	*Palmaria palmata*	Red	Chemical/enzymatic extraction	In vitro radical scavenging and chelating	Ghelichi, Sørensen, Náthia‐Neves, et al. 2024
	*Pyropia columbina*	Red	Enzymatic hydrolysis	Different in vitro assays	Cian et al. 2015
	*Porphyra dioica*	Red	Chemical/enzymatic extraction	Different in vitro assays	Pimentel et al. 2020
	*Colaconema formosanum*	Red	Sonication/enzymatic hydrolysis	In vitro radical scavenging	Windarto et al. 2024
	*Fucus spiralis*	Brown	Physical extraction/enzymatic hydrolysis	In vitro radical scavenging, chelating, and reducing	Paiva et al. 2017
Antihypertensive	*Gracilariopsis lemaneiformis*	Red	Enzymatic hydrolysis	In vitro ACE Inhibition	Deng et al. 2018
	*Sargassum mcclurei*	Brown	Enzymatic hydrolysis	In vitro ACE Inhibition	Zheng et al. 2020
	*Fucus spiralis*	Brown	Physical extraction/enzymatic hydrolysis	In vitro ACE Inhibition	Paiva et al. 2017
	*Laminaria digitata*	Brown	Enzymatic hydrolysis	In vitro ACE Inhibition	Purcell et al. 2022
	*Saccharina japonica* (formerly *Laminaria japonica*)	Brown	Enzymatic hydrolysis	In vitro ACE Inhibition	J. Wang et al. 2021
Antimicrobial	*Solieria chordalis*	Red	Enzymatic hydrolysis	In vitro cell viability assay	Hardouin et al. 2014
	*Ulva lacinulata* (formerly *Ulva armoricana*)	Green	Enzymatic hydrolysis	In vitro cell viability assays	Hardouin et al. 2016
	*Saccharina latissima* (formerly *Saccharina longicruris*)	Brown	Enzymatic hydrolysis	In vitro bacterial growth inhibition assay	Beaulieu et al. 2015
Antiobesity and antidiabetic	*Grateloupia elliptica*	Red	Chemical extraction	Cell viability and animal study	H. G. Lee et al. 2020
	*Palmaria palmata*	Red	Chemical extraction	In vitro metabolic enzymes inhibition	Ghelichi, Hajfathalian, et al. 2025
	*Porphyra* spp.	Red	Enzymatic hydrolysis	In vitro α‐amylase inhibitory activity	Admassu et al. 2018
	*Palmaria palmata*	Red	Alkaline extraction/enzymatic hydrolysis	In vitro DPP IV‐inhibition	Harnedy and FitzGerald 2013
Anti‐inflammatory and immunomodulatory	*Ulva* spp.	Green	Chemical extraction/enzymatic hydrolysis	Animal study and cell viability	Cian et al. 2018
	*Caulerpa cupressoides*	Green	Chemical extraction	Animal study	Da Conceição Rivanor et al. 2014
	*Alsidium triquetrum* (formerly *Bryothamnion triquetrum*)	Red	Chemical extraction	Animal study	Fontenelle et al. 2018
	*Caulerpa sertularioides*	Green	Chemical extraction	In vitro protein denaturation inhibition and membrane stabilization assays	Sanniyasi et al. 2023
Antiproliferative, antitumor, and anticancer	*Codium decorticatum*	Green	Chemical extraction	Cell line models and in vitro lactate dehydrogenase release assay	Senthilkumar and Jayanthi 2016
	*Saccharina japonica* (formerly *Laminaria japonica*)	Brown	Chemical extraction	Cell line models and in vitro assay	Chen et al. 2021
	*Ulva prolifera* (formerly *Enteromorpha prolifera*)	Green	Sonication/aqueous extraction/enzymatic hydrolysis	In vitro cell growth inhibition assay	Lin et al. 2022
	*Pyropia haitanensis*	Red	Mechanical extraction/sonication	In vitro cell growth inhibition assays	Mao et al. 2017
	*Saccharina japonica* (formerly *Laminaria japonica*)	Brown	Chemical extraction	In vitro cell line models and animal study	Wu et al. 2022
	*Saccharina japonica* (formerly *Laminaria japonica*)	Brown	Chemical extraction	In vitro cell line models and animal study	Du et al. 2022

It is important to exercise caution when linking the bioactive properties of seaweed extracts exclusively to peptides. Many studies do not involve the purification of peptides from crude extracts, meaning that the observed biological activities may be influenced by other components, such as carbohydrates and phenolic compounds as well as minerals and vitamins, or their interactions with proteins and peptides (Eladl et al. 2024). Therefore, to accurately attribute bioactivity to seaweed‐derived peptides, it is essential to validate the observed effects using purified peptides. To achieve this, researchers can employ preliminary purification techniques such as salting‐out and dialysis, followed by more targeted chromatographic methods, including size‐exclusion chromatography and ion‐exchange chromatography, for the fractionation and purification of bioactive peptides (Naseem et al. 2024a). Additionally, performing proteomic and peptidomics analyses on crude extracts or their fractions can provide valuable insights into the existing peptide sequences. Computational in silico screening of these sequences can help predict peptides with the highest bioactive potential. Once identified, these peptides can be chemically synthesized and subjected to biological evaluations to confirm their functional properties (Hajfathalian et al. 2025). Such a comprehensive approach, combining experimental purification, proteomic analysis, and computational predictions, can significantly enhance the reliability of findings and establish a clearer link between seaweed‐derived proteins and their health benefits, particularly in the context of obesity and diabetes management.

## 3 Challenges in Extracting Proteins and Peptides From Seaweed

Despite the promising potential of seaweed‐derived proteins and peptides, their extraction and utilization face multiple technical and biological challenges. The complex structure of seaweed cell walls, the interaction of proteins with other biomolecules, and the presence of antinutritional factors and potential toxins complicate efficient extraction, purification, and utilization. Additionally, digestibility, bioavailability, and allergenicity must be carefully considered to fully realize the nutritional and functional benefits of seaweed proteins. Addressing these challenges through advanced extraction techniques, improved purification strategies, and rigorous safety evaluations will be crucial for the successful integration of seaweed‐derived proteins into food applications.

### 3.1 Cell Wall

**FIGURE 1:**
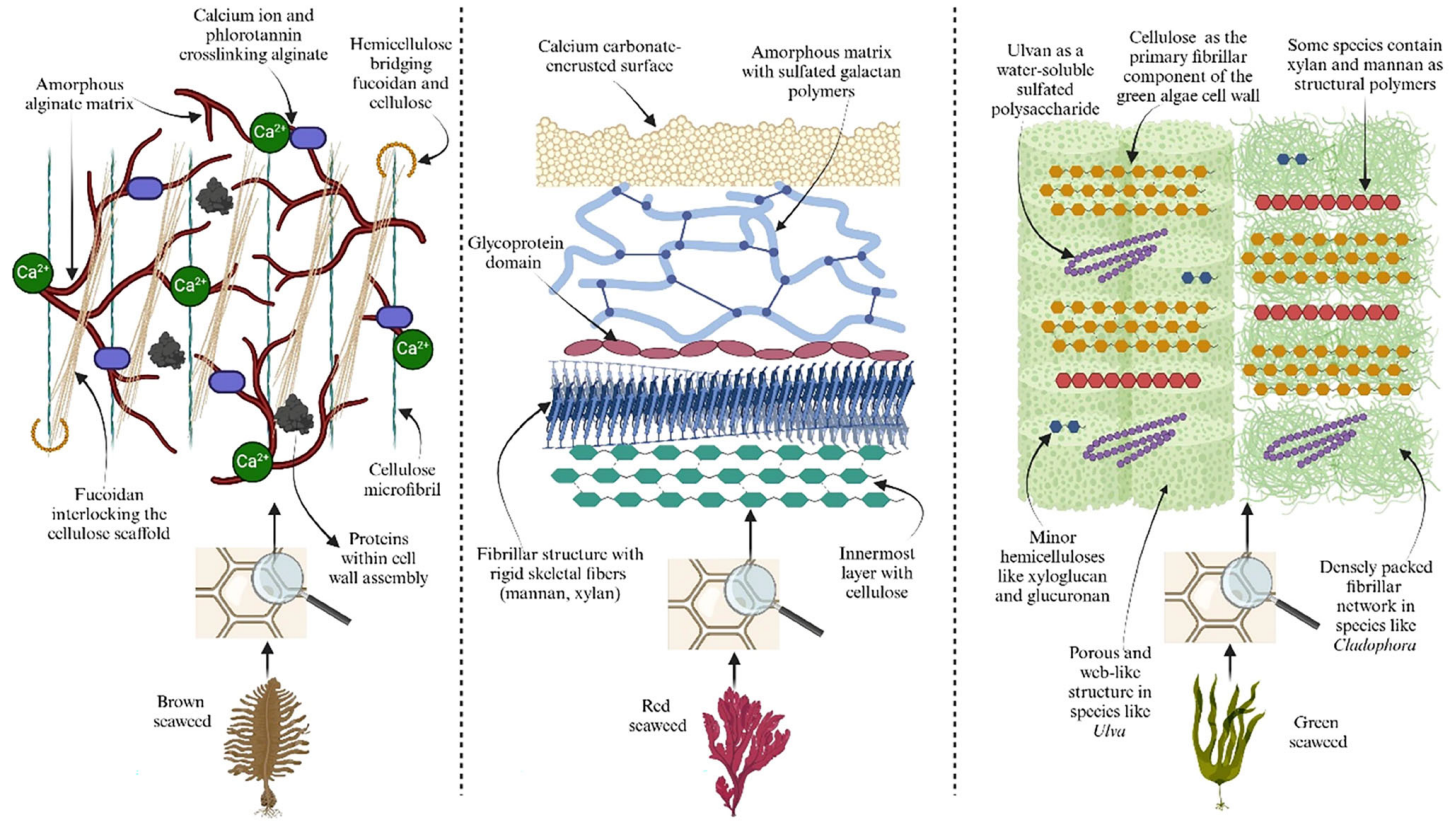
Cell wall composition of brown, red, and green seaweed (created in BioRender [https://BioRender.com/4g3i9ab]).

Despite the vast evolutionary differences between algae and land plants, both have developed polysaccharide‐rich cell walls that serve as structural barriers. While physically similar, the chemical composition of seaweed cell walls differs significantly from that of terrestrial plants, often containing unique sulfated polysaccharides, such as alginate and carrageenan, and ulvan (Fuertes‐Rabanal et al. 2024). Figure 1 illustrates the key structural and compositional elements of brown, red, and green seaweed cell walls. For a comprehensive review, readers are referred to Z. J. Lee et al. (2025). Here, a concise summary of the main cell wall components is provided based on their explanation:

- Brown seaweed: multilayered walls primarily composed of alginate and fucoidan, with a cellulose scaffold in an alginate matrix. Fucoidan links with cellulose, while alginate crosslinks with calcium ions and phlorotannins for rigidity. Phlorotannin–alginate complex reinforces the wall, with mixed‐linked glucans aiding assembly and elongation.
- Red seaweed: multilayered walls with three main components—(i) a rigid fibrillar network of cellulose, mannan, or xylan, (ii) an amorphous matrix rich in sulfated galactans such as agar, carrageenan, and porphyran, and (iii) a glycoprotein domain. Cellulose is a minor component or absent in some species, replaced by mannan and xylan. Coralline red algae contain lignin‐like structures for strength and deposit calcium carbonate on their outer surface.
- Green seaweed: walls like plant cell walls, primarily composed of cellulose. Some species contain xylan and mannan. In *Ulva*, cellulose forms a web‐like structure in a porous matrix, while *Cladophora* has a densely packed fibrillar matrix. The wall contains an amorphous matrix rich in ulvans, with minor hemicelluloses like xyloglucan and glucuronan. Outer structure varies by species, affecting mechanical properties.

Given the diverse and complex structures of seaweed cell walls across different species, extracting intracellular proteins requires a carefully balanced approach (Naseem et al. 2024b). The challenge lies in achieving both sustainability and efficiency, as the seaweed protein extraction process must be effective while maximizing sustainability and minimizing environmental impact (Pereira et al. 2024). Therefore, optimizing protein extraction techniques to maximize yield while ensuring sustainability remains a key focus in seaweed research and biotechnology.

### 3.2 Protein Solubility

As previously discussed, alkaline pH conditions are typically required to solubilize seaweed proteins. However, such alkaline environments can pose challenges in terms of consumer acceptance, particularly due to potential changes in taste, safety perception, or labeling concerns. At the same time, the protein extraction process must strike a careful balance between achieving a high protein yield, being cost‐effective and time‐efficient, and preserving the structural integrity of the extracted proteins (de Souza Celente et al. 2023). Many studies have employed the pH‐shift method, in which proteins are first solubilized under alkaline conditions, then precipitated by adjusting the pH to the isoelectric of the target proteins. At this pH, the proteins carry no net charge, resulting in reduced solubility and subsequent precipitation into a protein‐rich pellet (Thakur et al. 2024). Although this pellet contains a high concentration of protein, its poor water solubility limits its application in many food systems. Therefore, it is crucial to develop extraction strategies that improve protein solubility while also supporting consumer acceptance and ensuring process efficiency. An important consideration in developing these strategies is the need to maintain the extraction pH above the isoelectric point of the target proteins to ensure solubility, while avoiding excessively high pH levels that may compromise food safety, sensory quality, or consumer acceptance. Therefore, future research should focus on the application of alternative extraction techniques (discussed in the following sections) that avoid extreme pH conditions, aiming to maximize water‐soluble protein recovery under mild, food‐compatible conditions. Such approaches not only support high protein yield and functionality but also align with clean‐label and environmentally sustainable processing by minimizing the use of chemical additives for pH adjustment.

One often overlooked factor in the extraction of water‐soluble proteins from seaweed is the impact of using wet versus dried biomass. In many studies, seaweed is first air‐dried, oven‐dried, or freeze‐dried, then cut or pulverized before being rehydrated into a slurry for use as the substrate in protein extraction processes. However, the drying method can significantly influence the nutritional properties of seaweed and the efficiency of protein extraction, as it may alter the protein structure, cell wall integrity, or interactions with polysaccharides, ultimately affecting protein solubility and yield (Santhoshkumar et al. 2023). For instance, drying methods involving heat can lead to the degradation of nitrogenous compounds, resulting in the extraction of partially degraded proteins with altered solubility characteristics. Uribe et al. (2019) reported that all tested drying methods, except vacuum drying, led to the degradation of histidine, highlighting the sensitivity of certain amino acids to thermal processing. In contrast, freeze‐drying preserved protein integrity more effectively and yielded significantly higher levels of several amino acids, including aspartic acid, serine, glycine, arginine, threonine, alanine, tyrosine, valine, and cysteine, compared to vacuum drying, solar drying, and convective drying. Therefore, it represents a promising area of research to investigate the effects of different drying methods, in comparison to fresh biomass, on the yield of water‐soluble proteins extracted under pH conditions compatible with food applications.

### 3.3 Protein Purification

Another key challenge in seaweed protein extraction is obtaining a purified protein fraction, as the extract is often a mixture of proteins and various nonprotein compounds. Even with high protein yields, the extract typically includes carbohydrates, phenolic compounds, pigments, lipids, and minerals, all of which can affect protein properties (Echave et al. 2021). Moreover, antinutritional factors, toxic compounds, and potential allergens (discussed hereafter) may also be co‐extracted, which could pose risks to the safety and nutritional quality of the final product (Juel et al. 2024). However, protein purification from seaweed can be particularly challenging, as the extracted proteins are prone to forming covalent and noncovalent interactions with other biomolecules in the seaweed matrix, including carbohydrates, lipids, and phenolic compounds (de Souza Celente et al. 2023). These interactions may lead to the formation of protein aggregates that are difficult to dissociate using conventional separation methods. Covalent interactions, such as those between proteins and polysaccharides, can result in stable, irreversible complexes (Cermeño et al. 2020), while noncovalent interactions, such as hydrogen bonding or hydrophobic interactions, may lead to weaker but still significant protein associations (Ghelichi, Sørensen, Náthia‐Neves, et al. 2024). Therefore, a combination of various purification techniques may be more effective in enhancing the efficiency of seaweed protein purification. These methods range from general approaches such as ultracentrifugation, diffusion‐based separation, and salting‐out, to more targeted techniques like electrophoresis and chromatography. Readers are referred to Naseem et al. (2024b) for a comprehensive review of these methods in the context of seaweed protein purification. However, the application of these methods can be costly, particularly when considering scale‐up and industrial production of purified proteins from seaweed. Therefore, future research should focus on developing novel purification strategies that are both industrially feasible and cost‐effective. This represents a significant challenge in the broader application of seaweed as a viable protein source, especially in the food industry where cost and scalability are critical factors.

### 3.4 Digestibility

Although whole seaweed is a valuable source of protein with a nutritionally high‐quality amino acid profile, its digestibility in the human body is relatively low, which is primarily due to the presence of robust cell walls composed of water‐insoluble carbohydrates (e.g., cellulose) and water‐soluble polysaccharides (e.g., xylan and alginate) (Landeta‐Salgado et al. 2025). Therefore, extracting protein from the seaweed matrix is essential to enhance its digestibility. However, once intracellular proteins or hydrolysis‐derived peptides are released from the cell wall, they may interact with other components present in the extract, such as polysaccharides, phenolic compounds, and salts. These interactions can potentially compromise, or even worsen, the digestibility of the extracted proteins compared to their state when embedded within the intact seaweed matrix. For instance, Cebrián‐Lloret et al. (2024) reported that the digestible fraction from *Gelidium corneum* (Rhodophyta) contains higher levels of free amino acids compared to *Gracilariopsis longissima* (Rhodophyta), which is attributed to the higher agar content in the latter. This higher agar content may lead to saccharide–peptide interactions, reducing the release of free amino acids during digestion. This suggests that protein and peptide digestibility, even after extraction, may still be impacted by such interactions. Consequently, investigating the digestibility of seaweed protein is a valuable research area. Standardized in vitro digestion models, such as INFOGEST (Brodkorb et al. 2019; Minekus et al. 2014), can be employed to evaluate the bioaccessibility of seaweed‐derived nutrients across different forms, namely whole seaweed, crude protein‐rich extracts, and purified protein fractions, which may allow for a comparative assessment of how the structural context influences protein digestibility. The INFOGEST static in vitro digestion model simulates the human gastrointestinal process by sequentially mimicking the oral, gastric, and intestinal phases using physiologically relevant conditions such as pH, enzymes, electrolytes, bile salts, and digestion times, enabling the analysis of protein breakdown and the release of peptides and amino acids to provide insights into how effectively proteins are released and potentially absorbed during digestion (Demarco et al. 2022). Additionally, protein quality assessment methods based on amino acid scoring, such as PDCAAS and DIAAS (see Section 2.1), may be applied to extracted seaweed proteins and peptides to estimate their nutritional value in relation to human amino acid requirements (Landeta‐Salgado et al. 2025; Moreno et al. 2022; Reynolds et al. 2022). Together, these complementary approaches provide a more comprehensive understanding of the digestibility barriers associated with seaweed protein extraction and their implications for nutritional quality.

### 3.5 Antinutritional Factors, Toxic Compounds, and Allergenicity

Protein extraction from seaweed often yields protein‐rich extracts that also contain various co‐extracted compounds, some of which can act as antinutritional factors and compromise the nutritional quality and applicability of the proteins and peptides (Magnusson et al. 2019). For instance, compounds such as phlorotannins, polyphenols, lectins, saponins, and flavonoids, particularly when present in high concentrations, can interfere with protein digestibility and bioavailability (O'Brien et al. 2022). Therefore, careful consideration should be given to the presence and concentration of these substances during protein extraction and peptide production processes. Lectins, phytic acid, tannins, and polysaccharides in seaweed have been reported to impair protein digestibility and bioavailability, but methods like biomass pretreatment, enzymatic liquefaction, and fermentation can reduce these antinutritional factors and enhance protein quality (Salehi et al. 2019). Furthermore, it has recently been shown that pulsed electric field technology can enhance the absorption and utilization of plant‐based proteins by reducing antinutritional factors (Kheto et al. 2024). Therefore, it is crucial to understand how different extraction methods may either concentrate or reduce antinutritional factors in the resulting extracts. For example, Choudhary et al. (2023) analyzed 15 species of tropical seaweeds and found negligible levels of antinutritional compounds, including tannic acid, phytic acid, saponins, alkaloids, and terpenoids. However, it remains essential to assess whether protein extraction processes could lead to the accumulation of these antinutrients in the fractions where proteins and peptides are most concentrated. In this regard, the extraction method applied to obtain seaweed proteins and peptides also plays an important role.

Despite its rich nutritional profile, seaweed can contain toxic compounds that pose potential health risks. Limited attention has been given to their toxicity, which may arise from chemical compounds in the seaweed itself, epiphytic bacteria, harmful algal blooms, or absorbed heavy metals from seawater (Kumar and Sharma 2021). For example, a study on 37 different seaweed species, encompassing red, brown, and green types, revealed the presence of heavy metals such as cadmium, chromium, nickel, and vanadium (Oliveira et al. 2009). Minerals, especially iodine, at high concentrations can also be considered toxic compounds in seaweed and seaweed‐derived proteins and peptides. For example, a study reported that iodine levels in *Laminaria digitata*, *Laminaria hyperborea* (Phaeophyceae), and *Vertebrata lanosa* (Rhodophyta) were very high and that a daily intake of a small amount of these species (≤1 g) would exceed the tolerable limit for humans (1100 µg/day) (Mæhre et al. 2014). Excessive levels of toxic components in seaweed products could lead to harmful interactions with medications and disrupt hormone levels in the human body (Kumar and Sharma 2021). Research has shown that the presence and concentration of toxic compounds in seaweed depend on various factors such as seaweed type (red, brown, green), species, growth location, and seasonal differences. For instance, Jönsson and Nordberg Karlsson (2024) reported that among the species they analyzed, *S. latissima* and *A. esculenta* had notably higher levels of arsenic and cadmium, with *S. latissima* having the highest iodine levels. They also reported site‐specific variations in inorganic arsenic, lead, and cadmium in *S. latissima* from three different sites (Ireland, Norway, Sweden). Therefore, strategies should be implemented to reduce or ideally eliminate toxic compounds during protein extraction from seaweed. This requires careful selection of optimal treatments or pretreatments on seaweed biomass. For example, warm water and hypersaline solution soaking treatments have been shown to reduce iodine in *S. latissima* and cadmium in *A. esculenta*, respectively (Stévant et al. 2018). However, these treatments must be performed with care, and proper optimizations are necessary to avoid the loss of target compounds, such as proteins and peptides. For example, it has been recently reported that warm seawater treatment at 45°C, but not 35°C, for 1–2 min effectively reduced the iodine concentration in *A. esculenta* and *S. latissima* with favorable nutrient retention (Stévant et al. 2025). Therefore, efforts to extract proteins and peptides from seaweed should account for the risk of toxic compounds and be designed to ensure that the levels of these compounds remain below the danger threshold for human consumption, thereby overcoming this challenge.

Like antinutritional factors and toxic compounds, the allergenicity of seaweed protein and peptide products could also pose a significant challenge in their application for human consumption (Garciarena et al. 2022). Allergenicity has been a considerable concern regarding the use of alternative protein sources, necessitating special attention to avoid allergic reactions (Günal‐Köroğlu et al. 2025). Research on the allergenicity of seaweed protein and peptides is still in its early stages, with most studies focusing on the optimization and analysis of extracted proteins, particularly from microalgal species (James et al. 2023). Reports have indicated the allergenicity of seaweed proteins from brown seaweeds like *S. latissima* and *A. esculenta* (Mildenberger and Rebours 2025), red seaweeds like *P. palmata* (Garciarena et al. 2022), and green seaweed like *Ulva* sp. (Polikovsky et al. 2019). However, seaweed protein is generally considered safe in terms of allergenicity (Mildenberger and Rebours 2025). However, it is recommended that regular allergenicity studies be conducted on seaweed‐derived proteins and peptides to ensure the safety of this valuable alternative protein source.

## 4 Protein Quantification

To assess the efficiency of protein extraction from seaweed, it is essential to accurately quantify the protein content in the target fraction, where the extracted protein is expected to accumulate (Naseri, Marinho, et al. 2020). This quantification is typically coupled with mass balance calculations to determine protein yield, expressed as the amount of recovered protein relative to the initial biomass and its processed fractions (Templeton and Laurens 2015). Determining the protein content in seaweed extracts is not straightforward because of the presence of co‐extracted “impurities” like carbohydrates, phenolic compounds, and salts (Niemi et al. 2023). Protein quantification methods for seaweed can be broadly categorized into three main groups: spectroscopic techniques, amino acid analysis, and nitrogen content determination. Each of these approaches differs in terms of specificity, sensitivity, accuracy, and suitability for complex matrices like seaweed. Protein quantification methods for seaweed are briefly presented below, with a focus on the frequently discussed issue of the nitrogen‐to‐protein conversion factor in relation to seaweed protein quantification based on nitrogen content.

### 4.1 Spectroscopic Techniques

The spectroscopic methods to determine seaweed‐derived proteins and peptides include either colorimetric methods, such as Lowry, Bradford, or bicinchoninic acid (BCA), or label‐free methods, such as near‐infrared (NIR) and Fourier‐transform infrared (FTIR) spectroscopies. Although dye‐based methods are widely used due to their simplicity, speed, and cost‐effectiveness (Niemi et al. 2023), they may not provide accurate quantification of seaweed‐derived proteins and peptides. This limitation is primarily due to the complex matrix of seaweed, which contains interfering compounds such as polyphenols, pigments, and nonprotein nitrogen that can affect dye‐binding efficiency (Rawiwan et al. 2022). Nevertheless, assays such as Bradford and Lowry have been applied to estimate protein content in certain seaweed species, including *S. latissima* (Harrysson et al. 2018), *Ulva* sp. (Shuuluka et al. 2013; Steinbruch et al. 2024), and different red seaweed (Rawiwan et al. 2022), albeit often with caution regarding their accuracy and comparability to other quantification methods. Alternatively, label‐free spectroscopic methods have been recommended as a rapid and environmentally sustainable approach for quantifying protein content in seaweed (Niemi et al. 2023). For instance, NIR and mid‐infrared (MIR) spectroscopy methods have been reported to provide accurate quantitative prediction of crude protein in four brown seaweed species (*L. digitata*, *Ascophyllum nodosum*, *Fucus vesiculosus*, and *S. latissima*) (Campbell et al. 2022). Overall, spectroscopic approaches for determining the protein content of seaweed extracts offer advantages in terms of speed, simplicity, and cost‐effectiveness. However, both colorimetric and label‐free methods may be susceptible to interference from nonprotein components in the seaweed matrix and often lack specificity, which can compromise the accuracy of the measurements.

### 4.2 Amino Acid Analysis

Determining protein content by quantifying total amino acid content is another method used for seaweed extracts. This approach involves hydrolyzing proteins and peptides with a strong acid (e.g., 6 M HCl), followed by quantification of the total amino acids using mass spectrometry (Naseri, Jacobsen, et al. 2020). While this method provides an accurate estimation of protein content, it has several limitations, including the need for expensive equipment, significant time, chemicals, and specialized knowledge (Niemi et al. 2023). Additionally, the extreme acidic conditions during hydrolysis can degrade certain amino acids such as glutamine, asparagine, tryptophan, and cysteine, making them difficult to detect (Ghelichi, Hajfathalian, et al. 2025). Single hydrolysis may not ensure the complete release of all amino acids, potentially leading to underestimation (Angell et al. 2016), but extending the hydrolysis process could cause further destruction of amino acids before quantification. It is also recommended to account for the water gained during initial hydrolysis before quantifying amino acids to measure protein content accurately (Ghelichi, Sørensen, Hajfathalian, et al. 2024). Overall, while total amino acid quantification can reliably measure protein content in seaweed, the potential destruction and partial hydrolysis of certain amino acids, as well as the complexity of the method, should be considered.

### 4.3 Nitrogen Content Determination and Nitrogen‐to‐Protein Conversion Factor

The most common approach to analyze the protein content of seaweed and its extracts is via the determination of nitrogen content through methods like Kjeldahl or Dumas by considering a 16% nitrogen content of protein and a nitrogen‐to‐protein conversion factor of 6.25, which has been found to overestimate protein content in the case of seaweed (Bjarnadóttir et al. 2018). It has been suggested that due to the presence of nonprotein nitrogen in various biomasses, the most accurate method for true protein quantification based on nitrogen content involves using a nitrogen‐to‐protein conversion factor derived from quantitative amino acid composition (Shuuluka et al. 2013). This approach accounts for the specific amino acid profile of the biomass, providing a more precise estimation of protein content by distinguishing between protein‐derived nitrogen and nonprotein nitrogen sources. For example, Ghelichi, Sørensen, Hajfathalian, et al. (2024) reported that, based on total amino acid quantification considering the water gained during initial hydrolysis, the nitrogen‐to‐protein conversion factors are approximately 4.5 for dried seaweed and approximately 4 for seaweed extracts, while these figures are close to 5 when the water gained during the hydrolysis stage of amino acid analysis is not considered. Bak et al. (2019) also reported a significant variation in their calculated nitrogen‐to‐protein conversion factor from the commonly used 6.25, concluding that due to the variations and uncertainty regarding this conversion factor for seaweed, determination of protein content based on nitrogen content is not feasible. They alternatively suggested the use of amino acid quantification as a basis for protein content in seaweed. However, the latter also faces limitations and challenges, as mentioned in the previous section. In this regard, Angell et al. (2016) performed a meta‐analysis by analyzing the variation in nitrogen‐to‐protein conversion factor for 103 species across 44 studies from various phyla, geographic regions, and nitrogen contents, concluding that the conversion factor of 5 can be considered a “universal” conversion factor for seaweed. However, Biancarosa et al. (2017) recommended that this factor should be studied for different species of seaweed separately, as they found varying conversion factors from approximately 3.5 to 5 for different species of seaweed. This should be complemented by highlighting that this factor should be studied not only for each species differently but also for extracts obtained from a single species, meaning that the conversion factor applied for a single species cannot necessarily be applied to the resulting extracts. This might also apply to different extraction techniques, such as chemical extraction and enzymatic hydrolysis, which might necessitate applying different nitrogen‐to‐protein conversion factors due to varying effects of these techniques on the change or release of interfering compounds such as phenolic compounds or nonprotein nitrogenous compounds. Similarly, various pretreatment and drying techniques could also impact the true conversion factor in seaweed and resulting extracts. Therefore, future studies are recommended to analyze the amino acid composition of the biomass and resulting extracts by quantifying total amino acids and considering the water gained during the initial hydrolysis of amino acid quantification to calculate the actual conversion factor. This will allow future studies on the same species of seaweed, or on the extracts obtained using a similar extraction method, to refer to these results and use the conversion factors presented by these studies. However, it may be feasible to say that until the time detailed information on this factor is available for most seaweed species and various treatment techniques, the conversion factor of 5 could be used to obtain a near‐to‐real picture of protein content in seaweed and seaweed extracts.

## 5 Seaweed Protein Extraction Methods

This section provides a critical overview of the various protein extraction techniques used for seaweed. It is important to note that these methods will not be discussed in exhaustive detail, as they have already been thoroughly reviewed in several previous studies (e.g., de Souza Celente et al. 2023; Echave et al. 2021; Naseem et al. 2024b). Readers seeking an in‐depth explanation of the methodologies are encouraged to consult those comprehensive reviews. Instead, this article offers a concise summary of the key extraction techniques, accompanied by a critical evaluation of their applicability, efficiency, and limitations specifically in the context of seaweed protein extraction.

### 5.1 Chemical Methods

Chemical extraction methods, also known as solvent‐based techniques, utilize solvents to disrupt the seaweed cell wall and solubilize proteins (Michalak and Chojnacka 2015). After solubilization, two main strategies can be employed. The first approach involves adjusting the pH to a level that maximizes protein solubility, typically at values far from the protein's isoelectric point (Rawiwan et al. 2022). The second strategy uses pH‐shift (also known as isoelectric precipitation) methodology to bring the pH close to the protein's isoelectric point, resulting in protein precipitation (Veide Vilg and Undeland 2017). This process forms a protein‐rich pellet, or “cake,” which can be collected as the primary product (Naseri, Marinho, et al. 2020). Each approach presents its own challenges. In the first scenario, optimal solubility is usually achieved at an alkaline pH (approximately 8.5–9) (Ghelichi, Sørensen, Hajfathalian, et al. 2024). However, this pH range is not ideal for most food applications. When the pH is subsequently lowered during food formulation, such as in acidic products, proteins may precipitate and be lost from the system. Therefore, future research should aim to enhance protein solubility at pH levels compatible with food‐grade formulations. In the second scenario, although the pH‐shift method yields a protein‐rich pellet, the insolubility of these proteins may limit their utility in food applications. Furthermore, direct consumption of the pellet can raise concerns due to the presence of antinutritional factors, potential toxins, and allergens (Section 3.5), along with possible sensory drawbacks (Trigo et al. 2024). Additionally, the chemical extraction of seaweed proteins raises sustainability and environmental concerns (de Souza Celente et al. 2023). These solvents can be difficult to manage and may lead to nonspecific hydrolysis (or denaturation) of proteins, producing peptides with unpredictable sequences and structures, random peptide linkages, and the formation of modified amino acids, some of which may pose potential health risks (Gajaria and Mantri 2022). Depending on the intended application, these variations can either enhance or compromise the functional or nutritional value of the extract. Overall, chemical extraction techniques provide a relatively straightforward and cost‐effective means of isolating protein from seaweed. However, key challenges must be considered, including the environmental impact of solvent use, limited control over protein structure and composition, and solubility of the extracted proteins under food‐relevant conditions.

### 5.2 Mechanical Methods

Mechanical methods are generally applied as pretreatment steps in seaweed processing to disrupt the algal cell wall and obtain a more homogeneous substrate, thereby facilitating subsequent extraction processes (Echave et al. 2021). However, in a few cases, they have also been explored as standalone protein extraction methods, although with limited success. For instance, screw press extraction was applied as the sole method to recover protein from *Ulva fenestrate* (Chlorophyta), but it proved less effective than solvent‐based extraction in terms of both protein yield and solubility (Juul et al. 2021). In addition, bead milling, evaluated as an independent extraction method, yielded comparable protein quantities to solvent‐based approaches but showed lower selectivity for proteins, potentially leading to the co‐extraction of nonprotein components (Moldes et al. 2024). Despite these exploratory uses, mechanical techniques are predominantly employed as pre‐extraction treatments. A common example is the mechanical grinding or milling dried seaweed biomass, which is routinely performed before the main extraction step (Naseri, Marinho, et al. 2020). Although mechanical pretreatment is a standard part of most seaweed protein extraction workflows, its specific influence on extraction efficiency and protein quality remains underexplored and warrants further investigation.

### 5.3 Enzymatic Methods

Enzymatic hydrolysis of seaweed biomass is one of the most widely utilized methods, if not the most prevalent, for extracting proteins from seaweed. Several terms have been used to describe the process of using enzymes for protein extraction from seaweed, including “enzymatic hydrolysis,” “enzymatic extraction,” and “enzyme‐assisted extraction.” Despite the variety of terminology, the overarching approach of using enzymes to extract proteins from seaweed can be understood from two key perspectives. First, enzymatic extraction can be viewed in terms of the method's application: whether enzymes are employed as the sole treatment (Vásquez et al. 2019) or in combination with other techniques to enhance extraction efficiency (Naseri, Marinho, et al. 2020). Second, the use of enzymes can be considered in relation to the goal of the process. In one scenario, enzymes are used to maximize protein extraction yields, aiming to recover as much protein as possible from the biomass (Steinbruch et al. 2023). In another, enzymes are used not only to extract proteins but also to hydrolyze them into smaller peptides, which may possess specific capabilities like antioxidant properties (Ghelichi, Sørensen, Hajfathalian, et al. 2024).

A wide range of enzymes has been investigated for their effectiveness in extracting proteins from seaweed. These enzymes generally fall into two main categories of polysaccharidases and proteases. Numerous studies have explored the use of these enzymes, either individually or in combination (Choulot et al. 2025; Nova et al. 2024), reporting varying yields and properties of the extracted proteins. While enzyme‐assisted extraction of seaweed proteins has been extensively studied, a comprehensive review of this approach is beyond the scope of the present paper due to space constraints. Although several reviews have addressed enzyme‐assisted extraction from seaweed biomass (e.g., Rhein‐Knudsen et al. 2015; Sanjeewa et al. 2023; Wijesinghe and Jeon 2012), they typically encompass a broad spectrum of bioactive compounds, including polysaccharides and other nonprotein components. Given the critical role of enzymes in protein extraction specifically, a focused and detailed review dedicated to enzyme‐assisted protein extraction from seaweed is warranted to better evaluate and advance this method. Regarding polysaccharidases, their selection is assumed to be based on the specific carbohydrate composition of the target seaweed's cell wall (de Souza Celente et al. 2023). However, predicting enzyme efficacy solely based on cell wall composition remains challenging. For instance, although the cell wall of *P. palmata* is primarily composed of xylans (Xu et al. 2024), xylanase did not significantly enhance protein extraction efficiency compared to other polysaccharidases such as Celluclast and Viscozyme (Naseri, Marinho, et al. 2020). Furthermore, our recent study showed that in some cases, proteases alone can outperform polysaccharidases in terms of protein extraction efficiency (Ghelichi, Sørensen, et al. 2025). This suggests that effective protein recovery may depend on factors beyond polysaccharide degradation (de Souza Celente et al. 2023), such as the overall structural integrity of the cell wall or the specificity of the enzymes used. To achieve optimal protein yields and desired functional properties through enzyme‐assisted extraction, it is essential to tailor the enzyme strategy by considering the type, combination, and concentration of enzymes specifically to each seaweed species. This optimization requires a nuanced understanding of both the biochemical composition of the biomass and the mode of action of the selected enzymes.

### 5.4 Combined Methods

Various extraction methods are often applied sequentially to enhance protein recovery from seaweed including enzymatic and solvent‐based extraction (Ghelichi, Sørensen, Hajfathalian, et al. 2024; Naseri, Marinho, et al. 2020), mechanical and solvent‐based extraction (Juul et al. 2022), and mechanical and enzymatic extraction (Je et al. 2009). In addition, a range of emerging technologies has been explored either individually or in conjunction with traditional methods to improve protein extraction efficiency and influence the functional properties of the resulting proteins and peptides. These include ultrasound (Braspaiboon et al. 2022), microwave (Wijethunga et al. 2025), pulsed electric field (Robin et al. 2018), autoclave and high‐pressure processing (O'Connor et al. 2020), subcritical water (Polikovsky et al. 2020; Trigueros et al. 2021), and supercritical fluid (Sarkar et al. 2022). These methods offer several advantages, including improved extraction yields, reduced processing times, and, in some cases, enhanced functional or nutritional properties of the extracted proteins. Many of these methods are regarded as green and sustainable alternatives to conventional, chemical‐intensive techniques. However, challenges related to economic viability, processing time, extraction efficiency, protein digestibility, and sensory attributes must be carefully considered (de Souza Celente et al. 2023). Overall, it is premature to recommend a single procedure as the optimal strategy for seaweed protein extraction. The field still requires substantial development to better understand and tailor extraction methods to the unique characteristics of each seaweed species.

## 6 Seaweed Protein Extraction From Biorefinery Perspective

Despite the nutritional and bioactive potential of seaweed‐derived proteins and peptides, proteins typically constitute only a small fraction of the total biomass in most seaweed species. Regardless of classification (red, brown, or green), seaweeds contain a rich matrix of other valuable compounds, including polysaccharides, minerals, lipids, vitamins, pigments, and, in some cases, other protein‐like bioactives (Johnston et al. 2023). Focusing exclusively on protein extraction without considering these other constituents may lead to inefficiencies and sustainability concerns. From a biorefinery standpoint, such a single‐target extraction strategy is not optimal. The core principle of biorefinery is to maximize the value derived from all biomass components through integrated and economically viable processes, adopting a cascading approach that aligns with circular economy goals and minimizes waste (Torres et al. 2019). Seaweed biorefineries aim to valorize the entire biomass by enabling the sequential extraction and utilization of bioactive compounds, contributing to a zero‐waste paradigm while also reducing environmental impacts such as greenhouse gas emissions (Nilsson et al. 2022). In addition, seaweed holds a central position in the development of the Blue Economy, a sustainable ocean‐based economic model, and plays an increasingly important role in the EU Green Deal's strategy for reducing reliance on fossil‐based resources (Lange et al. 2020). Beyond food and nutraceuticals, seaweed biomass has demonstrated utility in diverse industries (Torres et al. 2019)—for instance, as fertilizers in agriculture (Civelek Yoruklu et al. 2022); as functional ingredients in animal feed (Mahrose and Michalak 2022); in pharmaceutical (Lomartire and Gonçalves 2022) and cosmeceutical (Kalasariya et al. 2024) applications; as bioadsorbents for wastewater treatment (Santoro et al. 2022); and in the production of biofuels (Soares Dias et al. 2023).

A feasible biorefinery strategy for seaweed protein extraction involves designing integrated processing pathways where the outputs or residuals from one process serve as feedstock for the next. In such a cascading system, valuable compounds such as polyphenols, polysaccharides, or pigments may be extracted in early stages, with the remaining protein‐rich fraction subsequently processed for protein and peptide recovery. Conversely, proteins and peptides may be extracted early, followed by valorization of the residual biomass for other applications, including bioenergy or materials production (Balina et al. 2017). The order of extraction is critical and depends on the stability and compatibility of the target compounds under different processing conditions. For example, it has been reported that in red seaweeds such as *Eucheuma denticulatum*, *K. alvarezii*, *Chondrus crispus*, *Sarcothalia crispata*, and *Furcellaria lumbricalis*, it is important to extract proteins before carrageenan (or furcellaran in the case of *F. lumbricalis*), as the conditions used for carrageenan (or furcellaran) extraction could denature and degrade proteins (Naseri et al. 2019). Furthermore, enzyme‐assisted extraction at neutral pH and room temperature from *E. denticulatum* has been shown to efficiently recover proteins without compromising the yield or quality of carrageenan obtained afterward (Naseri, Jacobsen, et al. 2020), thus maintaining the functional properties required for industrial applications. This circular approach ensures that the entire biomass is utilized effectively, minimizing losses and promoting economic and environmental sustainability. Therefore, two main strategies can be envisaged in the context of protein extraction to align with the biorefinery framework: (i) downstream protein extraction, where valuable nonprotein compounds (e.g., polysaccharides) are extracted first, and the remaining biomass, now relatively enriched in protein, is processed for protein and peptide recovery, and (ii) upstream protein extraction, where proteins and peptides are extracted first, particularly if high‐purity or bioactive peptides are the primary target, and the leftover biomass is then utilized for other purposes, such as extraction of residual polysaccharides, production of biogas, or use as agricultural feedstock. When high‐purity, water‐soluble proteins and peptides are desired, additional purification steps such as ultrafiltration or chromatographic separation techniques are necessary (Section 3.3). These purification stages may generate additional byproducts, which themselves can be valorized in the biorefinery system, further contributing to resource efficiency (Arias et al. 2025).

**FIGURE 2:**
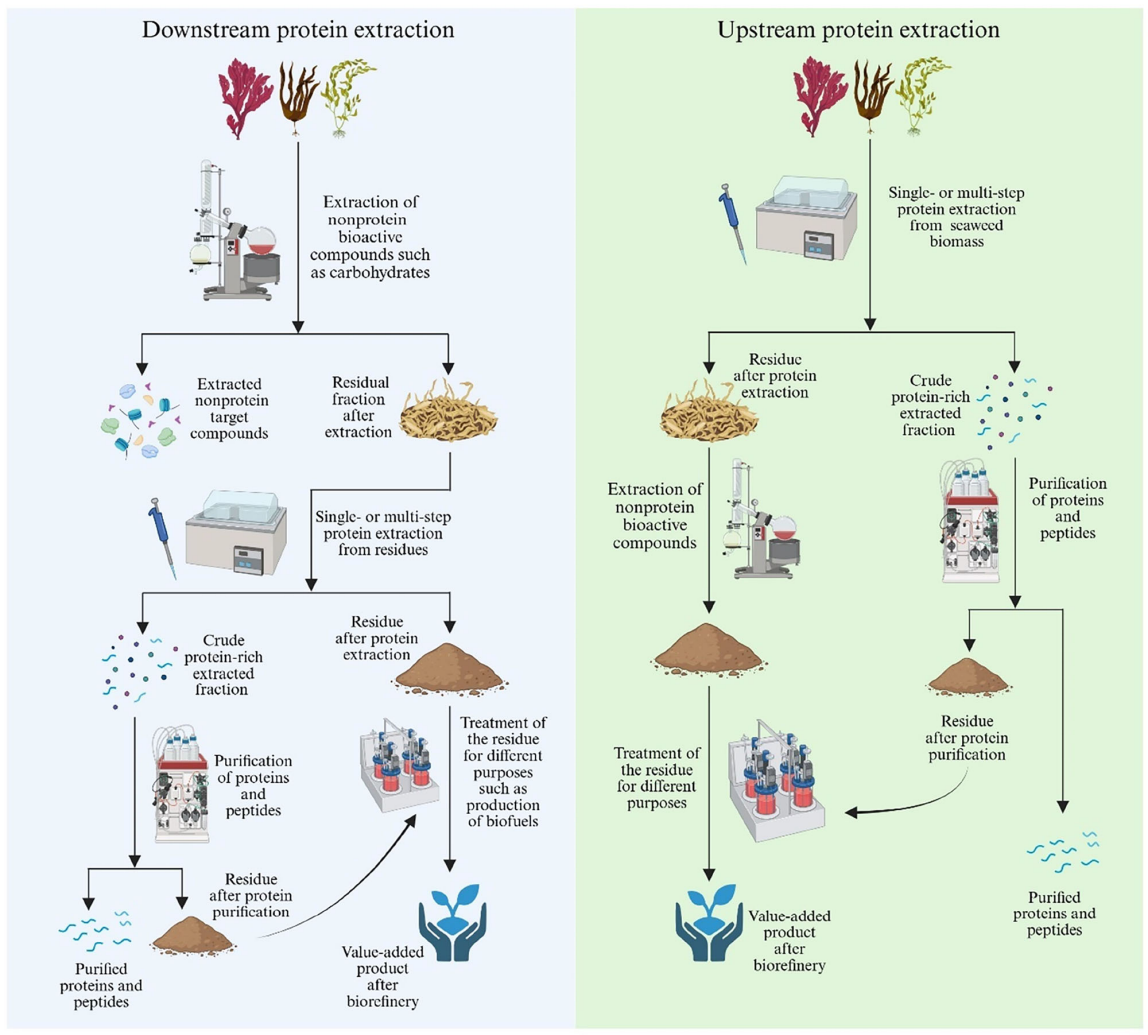
A conceptual schematic of seaweed protein extraction from a biorefinery perspective (created in BioRender [https://BioRender.com/vzbh2zw]).

Several biorefinery schemes for seaweed have already been proposed in the literature (e.g., Baghel 2023; Dickson and Liu 2019; Johnston et al. 2023; Meirelles et al. 2023; Nilsson et al. 2022; Seghetta et al. 2016). Figure 2 illustrates a conceptual schematic for seaweed biorefinery centered on protein extraction. This scheme is not intended to be definitive but may serve as a flexible framework that future studies can refine and adapt based on specific seaweed species, targeted products, and process conditions. Ultimately, this conceptual scheme is meant to provide a foundation for developing more economically viable and sustainable approaches to seaweed protein extraction that are in line with biorefinery and Blue Economy principles.

Overall, efforts to extract proteins and peptides from seaweed should not occur in isolation but rather be integrated into a comprehensive biorefinery framework. Such an approach emphasizes the full valorization of seaweed biomass, where proteins are just one of several valuable components to be recovered in a sequential and sustainable manner. By using the potential of co‐products, a biorefinery strategy not only enhances resource efficiency but also adds economic viability to the process, which is often a major hurdle in the commercial‐scale implementation of seaweed‐based technologies. This holistic model aligns with the principles of the circular economy and zero‐waste production, in which every output, whether a primary product or a residual stream, is seen as a potential input for further value creation. The cascading utilization of seaweed components enables industries to diversify product portfolios, reduce production costs, and minimize environmental impacts, particularly carbon emissions and marine waste. Moreover, integrating seaweed protein extraction into a biorefinery concept supports broader global sustainability goals, including those related to food security, clean energy, climate action, and responsible production. It also aligns with policy frameworks such as the EU Green Deal and the United Nations’ Sustainable Development Goals, further highlighting its relevance in the transition toward a more resilient and low‐carbon bioeconomy. To realize this vision, interdisciplinary research and industrial collaboration will be essential, focusing on process optimization, species‐specific valorization pathways, and scalable, energy‐efficient technologies. If successfully implemented, such integrated biorefinery systems can position seaweed as a key renewable resource in future food and bio‐based industries, solidifying its role as a cornerstone of a sustainable, circular blue economy.

## 7 Marketability and Commercial Development of Seaweed Protein

Despite scientific progress in characterizing the nutritional and functional potential of seaweed‐derived proteins, their commercial development remains in its nascent stage (Hajfathalian et al. 2025). To date, to the best of our knowledge, there are no widely marketed products that offer purified or concentrated seaweed protein isolates comparable to those from soy, pea, or other land‐based crops. Instead, seaweed proteins are predominantly present as minor constituents in blended products, such as plant‐based seafood alternatives, nutritional supplements, and functional health foods (Fleurence 2025), where seaweed's holistic nutritional profile that is rich in fiber, minerals, and bioactives is the primary appeal. Several technical and economic barriers have hindered the scalability of seaweed protein production. Protein content in seaweed is relatively low compared to its high polysaccharide composition, and the species‐specific complexity of seaweed cell walls poses a major challenge to efficient protein release (L. Wang et al. 2025). Extraction methods that overcome these structural hurdles such as enzymatic hydrolysis, high‐pressure processing, and pulsed electric fields are still largely confined to laboratory or pilot scale and can be cost‐prohibitive for commercial application (Russo et al. 2024). Additionally, issues related to protein solubility, taste, digestibility, and functional behavior in food matrices further complicate product development (Samarathunga et al. 2023). From a regulatory and safety standpoint, concerns such as heavy metal accumulation, allergenic potential, and lack of standardization in extraction and labeling also contribute to the current lag in commercial availability (Ali et al. 2025). These concerns demand thorough toxicological and functional characterization before seaweed proteins can be positioned as mainstream ingredients.

The growing consumer interest in sustainable, marine‐derived, and functional ingredients is gradually shaping a favorable commercial environment for seaweed protein. While only a handful of companies currently focus specifically on producing seaweed protein isolates, the field is gaining momentum. For instance, Oceanium is working on the development of innovative protein ingredients and isolates from seaweed, intended for use as nutrient‐rich components in functional food and beverage applications. Moreover, a growing number of startups and research collaborations are exploring formulations where seaweed serves as a functional protein source. Notably, plant‐based seafood analogs and fermented products are among the most promising early applications (Farid et al. 2024). Overall, the commercial landscape for seaweed‐derived proteins is still in its infancy but holds significant promise. Progress will depend on continued advancements in extraction and purification technologies, the integration of seaweed into scalable biorefinery systems, and the demonstration of functional and nutritional efficacy. In addition, clear regulatory frameworks and consumer education will be essential for broader market adoption. If these foundational challenges are addressed through strategic research, innovation, and investment, seaweed protein can emerge as a key contributor to future sustainable food systems.

## 8 Conclusion and Future Directions

Seaweed is increasingly gaining attention as a viable source of plant‐based protein, offering significant potential to reduce the carbon footprint and improve sustainability. Seaweed‐derived protein is nutritionally valuable due to its favorable profile of EAAs, making it an attractive alternative to traditional animal‐based proteins. However, the extraction of protein from seaweed presents several challenges. Seaweed rigid cell wall structure requires extensive processing and specialized equipment, which can limit the widespread industrial application of this protein source. Moreover, issues related to protein solubility, digestibility, and the presence of antinutritional factors, toxic compounds, and allergens remain concerns that must be addressed before seaweed‐derived protein can be utilized more broadly in food systems. While considerable research has focused on the extraction and characterization of proteins and peptides from seaweed, fewer studies have explored their applications in food products. It is crucial that future research also investigates the incorporation of seaweed‐derived proteins and peptides into everyday foods, assessing their sensory properties, digestibility, and potential health benefits. The successful inclusion of seaweed proteins in food products could open new markets for sustainable protein sources, contributing to global efforts to reduce the environmental impact of food production.

Future studies should focus on optimizing protein extraction processes based on biorefinery principles, which aim to maximize the value of all components within the seaweed biomass. A biorefinery approach would ensure that not only the proteins but also other valuable compounds such as polysaccharides are effectively recovered and utilized, reducing waste and increasing the sustainability of the extraction process. Sequential extraction methods targeting different bioactive compounds could serve as a potential solution to this challenge, allowing for the efficient recovery of proteins while preserving the nutritional integrity of other seaweed‐derived components. Additionally, there is a need for the adoption of bottom‐up approaches to predict the properties of peptides and proteins prior to experimental optimization. By simulating and modeling the structure and functionality of peptides, researchers can streamline the optimization process, reducing the need for extensive experimental trials. Another important consideration is the impact of the extraction process on protein functionality. Many existing extraction methods are invasive and difficult to control, which can result in the formation of protein aggregates or undesirable interactions between proteins and other biomolecules in the seaweed matrix. These interactions may lead to reduced protein functionality or unfavorable sensory attributes in the final product. In conclusion, while the potential for seaweed as a source of plant‐based protein is significant, further research is needed to overcome the technical challenges associated with protein extraction, improve the digestibility and solubility of extracted proteins, and ensure the scalability of these processes.

## Author Contributions

**Sakhi Ghelichi**: conceptualization, methodology, investigation, funding acquisition, visualization, resources, writing – original draft, writing – review and editing. **Charlotte Jacobsen**: conceptualization, methodology, investigation, resources, funding acquisition, writing – review and editing, supervision.

## Conflicts of Interest

The authors declare no conflicts of interest.
